# Evaluation of Circulating Tumor Cells and Related Events as Prognostic Factors and Surrogate Biomarkers in Advanced NSCLC Patients Receiving First-Line Systemic Treatment

**DOI:** 10.3390/cancers6010153

**Published:** 2014-01-21

**Authors:** Laura Muinelo-Romay, Maria Vieito, Alicia Abalo, Marta Alonso Nocelo, Francisco Barón, Urbano Anido, Elena Brozos, Francisca Vázquez, Santiago Aguín, Miguel Abal, Rafael López López

**Affiliations:** Translational Medical Oncology, Health Research Institute of Santiago (IDIS), Complexo Hospitalario Universitario de Santiago de Compostela (SERGAS), Trav. Choupana s/n 15706 Santiago de Compostela, Spain; E-Mails: laura.muinelo.romay@sergas.es (L.M.-R.); mariavieito@gmail.com (M.V.); alicia.abalo.pineiro@sergas.es (A.A.); marta.alonso.nocelo@sergas.es (M.A.N.); franciscojavier.baron.duarte@sergas.es (F.B.); urbano.anido@gmail.com (U.A.); elenamaria.brozos.vazquez@sergas.es (E.B.); franciscavazquezrivera@yahoo.es (F.V.); santiago.aguin.losada@sergas.es (S.A.); miguel.abal.posada@sergas.es (M.A.)

**Keywords:** non-small cell lung cancer, circulating tumour cells (CTC), prognostic biomarker, CellSearch

## Abstract

In the present study we investigated the prognostic value of Circulating Tumour Cells (CTC) and their utility for therapy monitoring in non-small cell lung cancer (NSCLC). A total of 43 patients newly diagnosed with NSCLC were prospectively enrolled. Blood samples were obtained before the 1st, 2nd and 5th cycles of chemotherapy and analyzed using CellSearch technology. Both CTC and CTC-related objects (not morphological standard or broken epithelial cells) were counted. At baseline 18 (41.9%) patients were positive for intact CTC count and 10 (23.2%) of them had ≥5 CTC, while CK positive events were found in 79.1% of patients. The group of patients with CTC ≥5 at baseline presented worse PFS and OS than those with <5 CTC (*p* = 0.034 and *p* = 0.008, respectively). Additionally, high levels of total CK positive events were associated with poor prognosis in the group of patients with <5 CTC. Regarding therapy monitoring, patients presenting increased levels of CTC during the treatment demonstrated lower OS and PFS rates. All these data supported the value of CTC as a prognostic biomarker and as a surrogate indicator of chemotherapy effectiveness in advanced NSCLC patients, with the additional value of analyzing other “objects” such as apoptotic CTC or CK fragments to guide the clinical management of these patients.

## 1. Introduction

Lung cancer is the first cause of cancer-related deaths, with non-small cell lung carcinoma (NSCLC) accounting for around 85% of lung cancer cases [1]. At diagnosis, approximately 70% of patients present advanced disease, for which curative therapy will not be available. Recent advances in the treatment of the so-called “oncogene addicted lung cancers”, with mutations in *EGFR* or *EML-ALK* translocation, have improved their prognosis, but only in a small percentage of patients [2,3]. Therefore, there is a large need of more specific and active targeted agents in association to specific tumour biomarkers that allow a better guidance and monitoring of patients over the course of therapy.

The critical role that circulating tumour cells (CTC) play in the metastatic spread of carcinomas is now widely recognized. CTC represent a “liquid” biopsy with great value as a tumour biomarker for personalizing and monitoring patient treatment [4]. Considerable technological efforts have been made over the past two decades to develop platforms that reliably identify and count CTC [5]. The semi-automated CellSearch platform (Veridex LLC, Raritan, NJ, USA) has been used to demonstrate the prognostic significance of CTC count in patients with metastatic breast, prostate, and colorectal cancers [6,7,8]. This technology is nowadays the only one approved by the American Food and Drug Administration (FDA, Silver Spring, MD, USA) for its clinical use. Detection of CTC using CellSearch depends on EpCAM expression by tumour cells. However the paradigm of epithelial to mesenchymal transition (EMT) as a predominant mechanism for tumour cell invasion and metastasis raises the possibility that not all tumour cells in the circulation will express epithelial markers [9,10,11]. In NSCLC patients, low CTC detection rates were described using CellSearch, probably due to heterogeneity in EpCAM expression [12]. Although there is a small percentage of NSCLC patients positive for CTC using this technology, the detection of high CTC levels at baseline has been related with poor survival rates [13,14]. While changes of CTC during treatment have not been widely studied, increased levels after chemotherapy have been described as a poor prognostic factor [14]. In these studies only the number of morphological intact CTC was taken into account (nucleated, positive for CK 8, 18 or 19 and negative for CD45). Although only the morphological intact CTC could form metastasis the presence of epithelial cell fragments could have an additional value for predicting the patient outcome.

The present study was conducted to support the clinical significance of CTC in patients with advanced NSCLC at first line of chemotherapy, and to explore the relevance of CTC-related objects in this group of cancer patients. 

## 2. Results and Discussion

### 2.1. Patient Demographics

In the present study, sequential blood samples from 43 advanced NSCLC patients were analysed with CellSearch system from September 2011 to April 2013 (Table 1). Inclusion criteria were the diagnosis of stage IIIB or IV NSCLC, confirmed by histology or cytology, non-amenable to radical surgery, ECOG Performance Status (PS) ≤2 and undergoing first line chemotherapy. All patients signed informed consent approved by the correspondent ethical committee. Mean follow-up was 7.6 months (range 0.07–19.07). At the time of analysis, 35 (81.4%) patients had experienced disease progression resulting in a median PFS of 5.95 months (95% CI, 4.53–7.37) and median OS of 7.65 months (95% CI, 5.95–9.35). This is less than those reported in clinical trials but reflects the reality of lung cancer in an unselected population in clinical practice. Their average age was 62.7 (range 40–83 years). Most of patients were stage IV at diagnosis (90.7%), had adenocarcinoma histology (72.1%) and received a platinum doublet (86%). A total of 40 patients received at least one administration of chemotherapy after baseline draw. The therapeutic regimens included cisplatinum and pemetrexed (34.9%), combinations between platines and taxanes or other platinum-based combinations (51.2%). 7% of patients, who had poor PS received monochemotherapy with docetaxel or pemetrexed or a combination of vinorelbine and gemcitabine. Finally, three patients could not receive any chemotherapy due to physical deterioration.

**Table 1:** Demographics of patients included in the study.

Factors	Subgroup	n	%
Average age at baseline		62.7 (40–83)	
Sex	Male	37	86
	Female	6	14
Smoker	Current smoker	19	44.2
	Prior smoker	15	34.9
	Never smoker	7	16.3
	Unknown	2	4.6
ECOG PS	0–1	30	69.8
	2	13	30.2
Histology	Adenocarcinoma	31	72.1
	Squamous-cell	12	27.9
Tumour stage	IIIB	4	9.3
	IV	39	90.7
Metastasis location	Bone	11	25.6
	Liver	5	11.6
	Supra-adrenal	9	20.9
	Pleural	16	37.2
	Others	5	10.6
QT treatment	CDDP-PEM	15	34.9
	CDDP-/CBDCA-	22	51.2
	GEM-VNB	3	7
	The best suportive care	3	7

### 2.2. CTC and CTC Related Objects at Baseline

**Figure 1:**
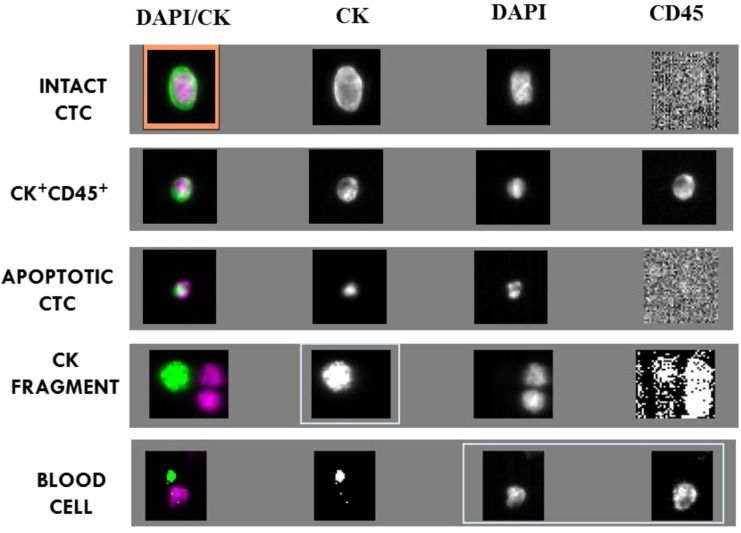
Images obtained after blood analysis of a NSCLC patient using CellSearch. We represented a morphological intact CTC (with an orange mark) round-oval, nucleated (DAPI^+^) >4 µm, CD45^−^ and CK^+^; a dual CK^+^ and CD45^+^ cell; an apoptotic CTC with CK^+^, CD45^−^ and DAPI^+^ staining but non standards of size and a CK fragment only with CK^+^ staining and a blood cell CD45^+^, CK^−^ and DAPI^+^.

Events upon CellSearch analysis were grouped in: morphologically intact CTC, CK^+^ and CD45^+^ cells, apoptotic CTC, CK fragments and blood cells (Figure 1). Tumour cells in circulation are considered as a heterogeneous population and their identification varies depending on the criteria used in each detection system. Intact CTC were considered as cells with nucleus >4 µm and CK^+^ CD45^−^ cytoplasm. Alternatively, cells possessing these features but dual CK/CD45 positive staining, were identified as CK^+^ and CD45^+^ cells. The origin of these cells is controversial, as leukocytes with positive CK or epithelial cells with CD45 expression. Apoptotic CTC were proposed as those events with CK and DAPI positive staining, but not morphologically accepted criteria [15], and CK^+^ fragments as events without DAPI staining. Finally, white blood cells, defined as cells with intact nucleus >4 µm and CK^−^ CD45^+^ cytoplasm, were also taken into account.

The average number of CTC and related objects and the percentage of positive events are summarized in Table 2. Morphologically intact CTC were present in 41.9% of patients at baseline with a mean of 18.9 CTC (range 0–637), with 23.2% of those patients having ≥5 CTC. The rate of positive test obtained in our study was in line with previous results using CellSearch technology (32%–40% of chemotherapy naïve stage IIIB/IV patients with detectable CTC), while the group of patients with high CTC count (≥5 CTC) was slightly higher [13,16]. Globally, the sensitivity of CellSearch system in NSCLC patients remains relatively low compared to other technologies such as Isolation by Size of Epithelial Tumour Cell (ISET) or CTC-Chip, with about 80% of CTC-positive cases [17]. Discrepancies between CTC reports using different technologies have been associated to the different isolation strategies, but a possible additional factor is that different methods could be accounting for different CTC subclasses.

To this regard, when CK positive events, intact or apoptotic CTC and CK fragments, were taken into account conjointly, we obtained a positive test in 79.1% of patients. On the other hand, CK^+^ and CD45^+^ cells were observed in 37.2% of patients. 

Additionally, apoptotic CTC and CK fragment levels positively and statistically correlated with intact CTC levels, being the strongest correlation between intact and apoptotic CTC (c = 0.7, *p* = 0.001, Spearman test), and suggesting a common origin (the primary tumour or metastasis) for all CK positive events. 

**Table 2:** Intact CTC and CTC-related object counts at baseline and over the course of therapy.

Type of Event	Baseline		2nd C		5th C	
	Mean ± SEM	Positive patients	Mean ± SEM	Positive patients	Mean ± SEM	Positive patients
INTACT CTC	18.9 ± 14.8	18 (41.9%)	0.87 ± 0.22	9 (31%)	0.77 ± 0.42	6 (28.6%)
CK^+^ CD45^+^ CELLS	2.6 ± 1.1	16 (37.2%)	ND	ND	ND	ND
APOPTOTIC CTC	8.4 ± 6.8	14 (32.6%)	10.57 ± 6.4	5 (17.2%)	3 ± 3	3 (14.3%)
CK FRAGMENTS	19.2 ± 9.6	32 (74.4%)	1.85 ± 0.61	12 (41.4%)	3.1 ± 1.35	14 (66.6%)
BLOOD CELLS	55 ± 20.48	43 (100%)	ND	ND	ND	ND

ND, non determined; SEM, standard error of the mean; 2nd and 5th C, second and fifth cycle of chemotherapy.

### 2.3. Correlation between CTC/CTC-Related Objects at Baseline and Clinico-Pathologic Features

We found a significant correlation between intact CTC levels at baseline and lymph node infiltration: patients with N3 stage (n = 23) were positive for intact CTC in a 58.3% *vs.* 22.2% observed in N0, N1, N2 stages (n = 19) (*p* = 0.029, Fisher’s test). Moreover, patients with at least two or more metastasis locations (n = 24) presented a higher ratio of CTC positivity compared to those with 0 or 1 metastasis (n = 18) (54.2% *vs.* 27.7%, *p* = 0.12 Fisher’s test). Finally, CK fragment presence was higher in adenocarcinomas (80.6% for adenocarcinoma and 54.5% squamous-cell tumours, *p* = 0.12, Fisher’s test).

These results confirmed in our series an increased spread of tumour cells and related artefacts concomitant to advanced disease. For NSCLC patients, high CTC levels were previously associated with the presence of several metastasis sites, presence of bone and liver metastasis and also with adenocarcinoma histology [13,18]. Interestingly, in our study the number of CK fragments was the only variable associated with histology. These CK fragments could be debris of epithelial cells with part of plasma membrane expressing EpCAM antigen, probably broken due to the blood flow. These fragments could be isolated by CellSearch because the method is not able to discriminate between nucleated and non-nucleated events.

### 2.4. Prognostic Significance of CTC/CTC-Related Objects at Baseline

**Figure 2:**
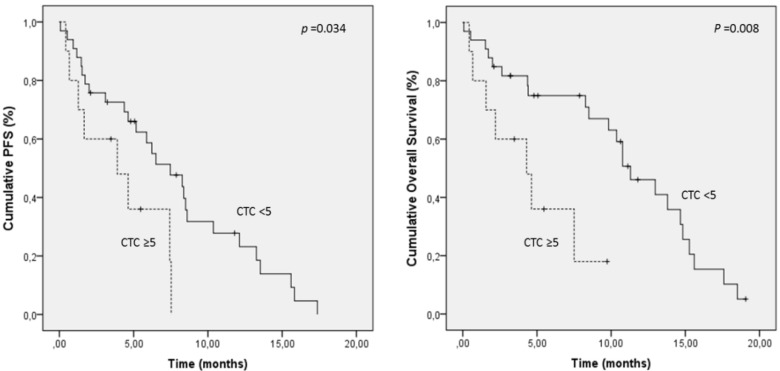
Kaplan-Meier curves for progression-free survival and overall survival of NSCLC patients grouping patients according to morphological intact CTC levels at baseline.

The clinical value of CTC as a surrogate biomarker relies on how consistently and accurately CTC can reflect tumour burden, prognosis and response to therapy. The possibility that CTC enumeration could stratify patients into prognostic subgroups with differential outcomes, and modify treatment plans to alter the course of NSCLC, would have an impact on patient management. In the present study, patients were categorized into favourable and unfavourable groups (5< *vs.* ≥5 morphologically intact CTC) for Kaplan-Meier analysis. Patients with CTC ≥5 presented a significantly shorter median PFS (4.1 months; 95% CI, 2.2–6) and OS (4.6 months; 95% CI, 2.5–6.8) compared to patients with CTC <5 (median PFS, 7.6 months; 95% CI, 5.7–9.5; median OS, 10.7 months; 95% CI, 8.6–12.8) (Table 3, Figure 2). The same cut-off has been previously described to discriminate patients with good and poor prognosis [5,13,16]. Krebs *et al.* examined the prognostic value of CTC enumeration in NSCLC patients employing CellSearch technology on stage III and IV patients, and described CTC counts ≥5 as a negative indicator for PFS and OS [13]. Other studies have also reported the prognosis value of CTC counts using CellSearch with cut-off thresholds of 1 or 2 CTC [16].

**Table 3:** Prognostic value of CTC and object levels at baseline.

Factor	Cut-Off	Median PFS	*p*	Median OS	*p*
INTACT CTC	5	4.1 *vs.* 7.6	0.034 *	4.6 *vs*. 10.7	0.008 *
APOPTOTIC CTC	2	3.4 *vs.* 7.6	0.017 *	3.6 *vs*. 10.5	0.001 *
CK FRAGMENTS	2	3.4 *vs.* 8.7	0.035 *	5.4 *vs*. 11.2	0.10
CK^+^ CD45^+^	2	6.5 *vs.* 6.8	0.59	7.3 *vs*. 9.6	0.86
BLOOD CELLS	17	6.7 *vs.* 7.1	0.60	9.1 *vs*. 9.6	0.97
INTACT/APOPTOTIC CTC	2	4.3 *vs.* 8.4	0.006 *	5.7 *vs.* 10.9	0.014 *
INTACT/APOPTOTIC CTC + CK^+^ FRAGMENTS	5	4.1 *vs.* 8.5	0.003 *	6.5 *vs.* 10.9	0.026 *

PFS, progression free-survival; OS, overall survival; * *p* ≤ 0.05.

Regarding CTC-related objects, CK^+^ CD45^+^ and white blood cells levels were not associated with survival (Table 3), consistent with previous works on prostate and breast cancers [19,20]. On the other hand, shorter survival was observed for the group of patients with high number of apoptotic CTC and CK fragments (Table 3). Interestingly, taking into account intact and apoptotic CTC or intact/apoptotic CTC and CK fragments together, PFS prediction power was improved (Table 3). In this sense patients with <5 intact CTC at baseline could be identified as patients with poor prognostic when they had ≥ 5 CK positive events (intact/apoptotic CTC and CK fragments together) (3.7 months; 95% CI, 1.4–6 *vs.* 8.6; 95% CI, 6.4–10.7 for PFS).

In addition, univariate Cox regression analysis of clinical factors showed that both standard CTC and related events such as apoptotic CTC and CK fragments were predictors for PFS together with the presence of bone metastases (Appendix Table A1). On the other hand, intact CTC and related events together with ECOG PS status and the presence of bone disease were found as OS predictors. Importantly, the parameters obtained taking into account intact and apoptotic CTC or intact and apoptotic CTC with CK fragments also showed prognosis relevance for both PFS and OS after univariate Cox regression analysis (Appendix Table A1).

In multivariate analyses including bone metastases, intact CTC and related events, we found that levels of intact CTC were an independent predictor for PFS while for OS the presence of bone metastasis is the only independent prognostic factor. The same analysis was applied for the combination of intact/apoptotic CTC or intact/apoptotic CTC with CK^+^ fragments, finding predictive power for the last group and only for PFS (Table 4). 

With these results, we demonstrated the impact in patient outcome of intact CTC, but also the important information about tumour burden that fragments and not standard cells may provide. This is especially relevant in tumours such as NSCLC where the rate of CTC detection is low [13,16]. Coumans *et al.* proposed that tumour microparticles are better indicators of the disease magnitude in castration resistant prostate cancers than CTC because they occur at higher frequency [20]. Similar results were described in renal cancer using CellSearch and in NSCLC using a fluid phase biopsy approach [21,22,23], however, to our knowledge, this is the work where the clinical value of CTC-related objects, isolated with CellSearch technology, has been demonstrated for NSCLC.

**Table 4:** Multivariate Cox regression analysis.

Factor	PFS		Factor	OS	
	HR (95%CI)	*p*		HR (95%CI)	*p*
Bone mets (no *vs*. yes)	2 (0.84–5)	0.11	Bone mets. (no *vs.* yes)	2.6 (1–6.5)	0.038 *
Intact CTC (<5 *vs.* ≥5)	4.3 (1.3–14.4)	0.016	ECOG PS (≤1 *vs.* 2)	2.1 (0.9–4.6)	0.071
Apoptotic CTC (<2 *vs*. ≥2)	1.3 (1.1–6.2)	0.16	Intact CTC (<5 *vs.* ≥5)	2.9 (0.7–11.4)	0.11
CK+ fragments (<2 *vs.* ≥2)	1.9 (1.2–4.8)	0.15	Apoptotic CTC (<2 *vs.* 2)	1.68 (1.13–4.4)	0.064
Bone mets. (no *vs.* yes)	2.4 (1.1–5.5)	0.028 *	Bone mets. (no *vs.* yes)	2.5 (1.2–5.6)	0.029 *
Intact/apoptotic CTC (<2 *vs.* ≥2)	1.4 (0.69–2.9)	0.33	ECOG PS (≤1 *vs.* 2)	2.5 (1.2–5.5)	0.016 *
			Intact/apoptotic CTC (<2 *vs.* ≥2)	1.3 (0.6–3)	0.45
Bone mets. (no *vs.* yes)	2.2 (0.9–4.9)	0.052			
Intact/apoptotic CTC+ CK^+^ fragments (<5 *vs*. ≥5)	3 (1.4–6.5)	0.005 *	Bone mets. (no *vs*. yes)	3 (0.96–5.5)	0.61
			ECOG PS (≤1 *vs.* 2)	2.3 (1–5)	0.029 *
			Intact/apoptotic CTC+ CK^+^ fragments (<5 *vs*. ≥5)	2.3 (0.9–5.7)	0.07

PFS. Progression Free Survival; OS. Overall Survival; HR. Hazard Ratio; CI. Confidence Interval; ECOG. Eastern Cooperative Oncology Group; PS. Performance Status. * *p* ≤ 0.05.

### 2.5. Changes of CTC/CTC-Related Objects within the Treatment

Advanced NSCLC lacks validated pharmacodynamic biomarkers for treatment response evaluation, usually analysed by radiologic imaging [23]. Thus, a simple and dynamic test, such as CTC analysis, which could early predict whether patients are benefitting or not from treatment, sooner than radiologic test, would be invaluable. With this objective, we analysed CTC levels at 2nd and 5th cycle of therapy compared to the baseline levels.

**Figure 3:**
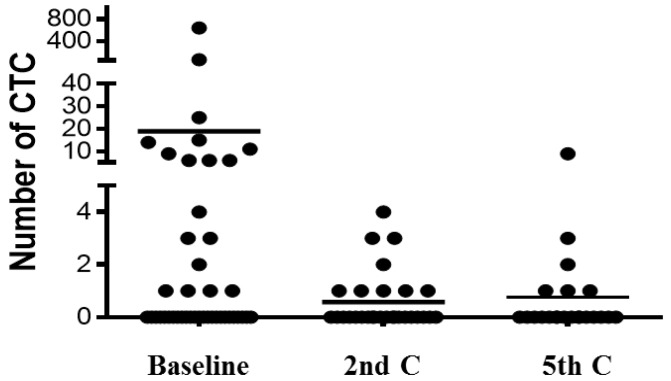
CTC levels at baseline, 2nd and 5th cycle of chemotherapy.

**Figure 4:**
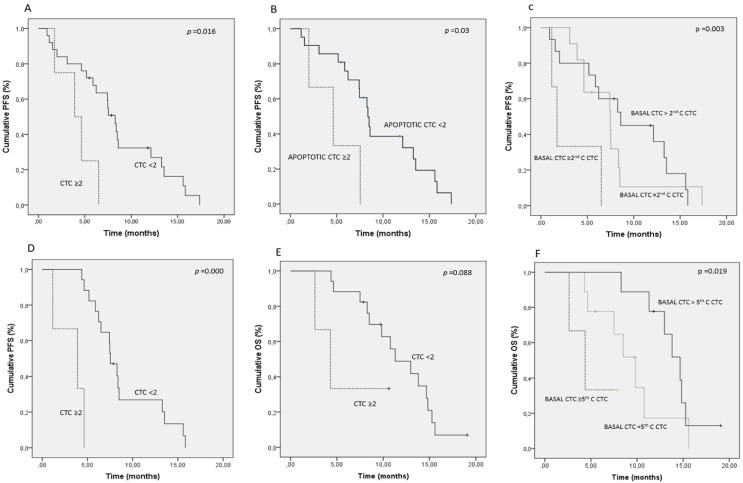
Kaplan-Meier curves for progression-free survival and overall survival. Only parameters with significant impact in patient outcome are represented. (**A**) Morphological intact CTC levels at 2nd cycle of chemotherapy; (**B**) CTC fragments at 2nd cycle of chemotherapy in patients with ≤2 CTC; (**C**) Changes in morphological intact CTC levels between baseline and 2nd cycle of chemotherapy; (**D**,**E**) Morphological intact CTC levels at 5th cycle of chemotherapy for PFS and OS, respectively; (**F**) Changes in CTC levels between baseline and the 5th cycle of chemotherapy.

We first observed a dramatic decrease in CTC levels at the 2nd cycle of chemotherapy in the majority of patients (Table 2, Figure 3), suggesting an overall response to therapy. For this analysis point, we redefined the CTC threshold because of the low number of CTC detected in almost all patients. In our opinion, this threshold, although low, is robust enough to discriminate between patients with poor or good prognosis because of the high sensitivity and reproducibility of the CellSearch technology. In fact, previously mentioned, cut-off thresholds of 1 or 2 CTC were used by other authors to define the group of patients with favourable outcome in NSCLC [16]. In the present work, patients with <2 CTC presented a significantly longer PFS (8.5 months; 95% CI, 6.4 to 10.5) compared to patients with ≥2 CTC (4.2 months; 95% CI, 2.2 to 6.1) (Figure 4A). Interestingly, patients showing an increase of intact CTC after cycle 1 of chemotherapy presented higher rates of progressive disease at radiological evaluation and, therefore, poorer PFS (Figure 4C). 

On the other hand, levels of CTC-related objects also suffered an important decrease after one cycle of chemotherapy. At this point, apoptotic CTC and CK fragments maintained their association with PFS, while changes between baseline and 2nd cycle of chemotherapy were not correlated to patient outcome. Even more important is the fact that patients with <2 intact CTC could be segregated into a group of poor and good PFS taking into account the CTC fragments (Figure 4B).

Finally, the prognostic value of morphological intact CTC was maintained at the 5th cycle of treatment using a 2 CTC cut-off (Figure 4D,E), while CTC-related events were not associated with patient outcome. Likewise, patients showing increased CTC levels before 5th cycle compared to baseline, presented poor PFS and OS (Figure 4F). The same trend was observed when we compared 2nd and 5th cycles (without statistical significance). 

Although all these results should be interpreted in the context of a small sample size, and further confirmed in a validation study, we demonstrated the potential of the CTC count as a surrogate endpoint for treatment follow-up. Taking into account that this evidence has been described in very few studies until now [13,14], our results presented relevance to demonstrate the clinical value of on-treatment evaluation of CTC in NSCLC patients. Notably, we also proved the utility of introducing the analysis of CTC-related objects together with the analysis of morphological intact CTC for prognosis and therapy monitoring. 

## 3. Experimental

### 3.1. Study Design

The present study was a prospective and single-centre study, developed at the Complexo Hospitalario Universitario de Santiago de Compostela, Spain. A total of 43 patients newly diagnosed with advanced NSCLC were enrolled. Inclusion criteria included radiologically confirmed stage IIIB/IV disease, ECOG performance status 0–2 and age higher than 18 years. Patients with a history of prior malignancy, within the previous 5 years, were also excluded. All patients gave written, informed consent to the ethically approved protocol. Clinical data were collected regarding age of the patient, smoking status, histology, TNM staging according to AJCC 7.0 and sites of metastatic disease (Table 1). Blood samples were obtained for analysis before the1st, 2nd and 5th cycle of chemotherapy.

### 3.2. CTC Analysis

CTC analyses were performed using CellSearch (Veridex LLC, Raritan, NJ, USA) technology. Samples were drawn into 10 mL evacuated blood drawtubes (CellSave, Veridex LLC), maintained at room temperature and processed within 96 h of collection. Cells expressing EpCAM were immunomagnetically enriched with the CellSearch system, from 7.5 mL of blood and fluorescently labelled with DAPI, CD45-APC, and CK-PE. Then the images of stained cells were acquired by a semiautomatic fluorescence microscopy system. Finally, two experimented reviewers selected the morphological intact CTC, defined as round-oval morphology, size more than 4 µm, nucleated (DAPI^+^), lacking CD45 and expressing CK, from the gallery of objects proposed by the system. In addition to the standard CTC, other objects such as CK positive fragments (with or without nucleus), were counted to determine their value for the clinical management of patients (see Figure 1).

### 3.3. Statistical Analysis

Correlation of CTC/objects counts with clinical variables was assessed by contingency table analysis using Fisher’s exact test. Correlations between baseline CTC/objects levels were compared using Spearman’s Rho analysis. The U-Mann-Whitney test was used to analyse CTC/objects counts within the treatment. PFS and OS were defined as the time elapsed between the date of baseline blood sample and the date of confirmed clinical progression, death, or censoring at last follow-up. Kaplan-Meier plots were developed for survival analysis and Log-rank tests were used to compare survival rates between groups. Univariate and multivariate Cox regression analyses were used to obtain HRs for the different groups with the appropriate 95% confidence intervals (CIs). To determine the most appropriate CTC/objects cut-off we used ROC curve analysis to establish the value that group the patients with early and late progression with high sensitivity and specificity. All statistical analyses were performed using SPSS 19 for Windows where *p* values of ≤0.05 were considered significant.

## 4. Conclusions

In summary, we report for the first time in advanced NSCLC patients the utility of the analysis of CTC-related events together with evaluation of morphological intact CTC with CellSearch technology as prognostic factors and surrogate biomarkers for treatment efficacy. Although these data require prospective validation in a larger series of patients, they provide robust evidences of the clinical value of CTC analysis for the management of this group of oncologic patients.

## Appendix

**Table A1:** Univariate Cox regression analysis.

Factor	PFS		OS	
	HR (95%CI)	*p*	HR (95%CI)	*p*
Age (<65 *vs*. ≥65 years)	1.36 (0.6–2.7)	0.38	2 (0.93–4.4)	0.07
Sex (male *vs.* female)	2.62 (0.8–8.7)	0.11	3.2 (0.7–13.6)	0.11
ECOG PS (≤1 *vs*. 2)	1.9 (0.94–3.9)	0.07	2.4 (1.1–5.2)	0.022 *
Histology (adeno. *vs*. squamous)	0.7 (0.36–1.7)	0.55	0.8 (0.34–1.9)	0.62
Stage (IIIB *vs*. IV)	2.5 (0.7–8.3)	0.13	4 (0.9–17.4)	0.057
N° mets. (≤1 *vs*. ≥2)	1.4 (0.7–2.9)	0.29	1.58 (0.7–3.3)	0.22
Bone mets. (no *vs*.yes)	2.4 (1.1–5.3)	0.038 *	2.6 (1.1–6.1)	0.026 *
Liver mets. (no *vs.* yes)	1 (0.3–3.5)	0.94	1.6 (0.47–5.8)	0.40
Pleural mets. (no *vs.* yes)	0.5 (0.25–1.2)	0.13	0.7 (0.36–1.6)	0.51
Supra-adrenal mets. (no *vs.* yes)	1.8 (0.8–4.1)	0.16	1.4 (0.6–1.4)	0.42
Baseline intact CTC (<5 *vs.* ≥5)	2.4 (1–5.7)	0.043 *	3.1 (1.2–8.2)	0.016 *
Baseline CK^+^ CD45^+^ (<2 *vs.* ≥2)	0.6 (0.25–1.6)	0.35	0.6 (0.3–1.5)	0.31
Baseline apoptotic CTC (<2 *vs.* ≥2)	1.8 (1.2–5.5)	0.025 *	2.6 (1.2–8.3)	0.009 *
Baseline CK^+^ fragments (<2 *vs.* ≥2)	2 (1.3–4.4)	0.036 *	1.7 (0.8–3.9)	0.15
Baseline Blood cells (<17 *vs*. ≥17)	0.8 (0.39–1.6)	0.51	1.1 (0.5–2.4)	0.78
Baseline intact/apoptotic CTC (<2 *vs.* ≥2)	1.9 (0.9–2.9)	0.016 *	2.3 (0.6–2.8)	0.021 *
Baseline intact/apoptotic CTC+ CK^+^ fragments (<5 *vs.* ≥5)	3.1 (1.5–6.8)	0.003 *	2.7 (1.1–6.6)	0.025 *

PFS: Progression Free Survival; OS: Overall Survival; HR: Hazard Ratio; CI: Confidence Interval; ECOG: Eastern Cooperative Oncology Group; PS: Performance Status. * *p* ≤ 0.05.
